# Patterns of use of oral health care services and barriers to dental care among ambulatory older Chilean

**DOI:** 10.1186/s12903-016-0329-2

**Published:** 2017-01-09

**Authors:** Rodrigo Mariño, Rodrigo A. Giacaman

**Affiliations:** 1 Oral Health Cooperative Research Centre Melbourne Dental School, University of Melbourne, Melbourne, VIC 3010 Australia; 2 Department of Oral Rehabilitation, University of Talca, Talca, Chile

**Keywords:** Oral health services utilization, Older adults, Chile

## Abstract

**Background:**

This paper describes the patterns of use of oral health care services among ambulant 65-74 years or older adults, living in the Maule Region of Chile, factors associated with their use of oral health care services, and self-reported barriers to using oral health care services.

**Methods:**

Four hundred and thirty eight older adults, aged 65–74 years, living independently in the community were orally examined and underwent an oral health interview. Recency of visits was related to the use of oral health care services within the 12 months prior to the study.

**Results:**

31.5% of respondents had used oral health services in the previous 12 months. In multivariate analyses, those living in rural areas (*OR =* 2.15; 95% CI:1.27–3.63), and those with secondary or higher education (*OR =* 1.65; 95% CI:1.03–2.64) visited the dentist in the last 12 months in a higher proportion. Those with more filled tooth-surfaces were more likely to have visited the dentist (*OR =* 4.02; 95% CI;3.58–4.51). Participants who self-reported dental fear, were less likely to have visited the dentist than those who did not (*OR =* 0.43; 95% CI;0.24–0.76).

**Conclusion:**

Comparing with existing data in Chile, participants in this study appear to have a slightly lower attendance. Findings question assumptions regarding oral health services utilization by rural residents and highlight the need to identify factors that influence the use of oral health services by older Chileans.

**Electronic supplementary material:**

The online version of this article (doi:10.1186/s12903-016-0329-2) contains supplementary material, which is available to authorized users.

## Background

Developments in medicine, improved social and economic conditions, in the Chilean population, have resulted in improvements of oral health, and, therefore, tooth retention, particularly, among Chilean older adults [1, 2], generating a growing need for them to seek regular oral health care. In addition, an increase in the life expectancy of the population, and its geographic distribution have resulted in an increased proportion of older people living in rural localities, with these regions ageing more rapidly than their counterparts in metropolitan areas [3]. This epidemiological and demographic transition has dramatically changed the nature of oral health care needs and it poses an oral health challenge. This may result in increased inequalities in access to oral health care services and in oral health outcomes, particularly among those who reside in rural areas [4, 5]. Such inequity in access to and utilization of oral health care services poses a major threat to the health and oral health of older Chileans.

Poor oral health impacts on the well-being and overall quality of life [6]. Adequate access to oral health care services becomes essential, as it provides opportunities for health promotion and disease prevention and early diagnosis, and treatment of oral diseases and conditions. Additionally, access to health care services is beneficial for the maintenance of good oral health, and health educational and awareness [7]. This is particularly the case when considering older populations. An equitable use of health care by the population is one of the most important goals of any health care system and is a human right. Consequently, achieving and maintaining the oral health of older adults is a major challenge for the Chilean health system and the society at large.

In 2007**,** the Chilean government implemented the Explicit Health Guarantee (GES) for those 60-years and older to ensure that their access to health is independent of their ability to pay [3, 8]. The GES includes 56 health conditions which are treated at no cost for the older population, including comprehensive oral health care, and some oral health conditions such as tooth loss and dental emergencies treatment for the whole population [8]. However, some health conditions are restricted to specific ages. For example, oral health care through GES is for 60 year-olds only [8].

Factors associated with the use of oral health services by older adults include: age, sex, level of education, social support and connectedness, income, general and oral health status, oral health knowledge and attitudes, perceived oral health needs, oral pain or discomfort, physical disabilities and chronic conditions [9–13]. Among older adults, underutilization of oral health services is common, with the most commonly cited barriers including the cost of dental care, shortage of professionals, and lack of familiarity with services provided and location of facilities [14, 15].

However, information on the oral health needs and oral health visit patterns of Chilean older adults is limited [8, 16, 17]. The national health survey reports that those aged 65 years and older account for 7.5% of dental visits [8]. Moreover, the data do not offer a clear picture on the use of oral health care services in rural populations, particularly those aged 65 and older. Because planning of oral health care services is dependent on the quality of current local data [18], as part of a larger study to assess the oral health profile of the Chilean Maule Region, this paper examines the patterns of use of oral health care services; factors related to the utilization of oral health care services, and barriers to using oral health services reported by in an ambulant population of older adults (65 to 74 years old) from the Maule Region. This information is necessary for adequate service planning and delivery to ensure the best possible oral health care for all Chileans. The influence of oral health on the quality of life of older adults and on the aging process, emphasizes the importance of exploring the use of oral health care services by older adults to better understand the socio-cultural context through which oral health decisions are made [19]. Providing specific information on regional populations will generate a robust advocacy instrument to argue for more oral health care resources; and place oral health within the broad concept of personal and social well-being.

## Methods

A community-based cross-sectional survey was selected. In the first stage, municipalities classified as urban or rural, were randomly selected from each strata, according to population size. In the second stage, individuals participating in third age clubs and other locations where older adults gather, were selected within each municipality. Power analysis was based on the case of 15 independent variables accounting for 10% of the variance in the dependent variable, with a sample size of 437 yielding a power of 0.99, that is an 99% chance of detecting an effect of that size at the level of significance of 0.01 [20].

After approval from the University of Talca Bioethics Committee, older adults’ social clubs members were invited to participate in the study. Once an individual consented to participate, he/she was asked to go through a structured interview and an oral examination at the clubs’ facilities. The methods used in this study have been previously described [2]. The interview covered a range of socio-demographic characteristics and use of oral health care services. The interview also included questions about attitudes to oral health and knowledge of causes and risk factors for dental caries, periodontal disease and oral cancer adapted from previous studies [21–23]. The instrument used in this study is included as “Additional file 1”. Clinical examinations were conducted by four trained and calibrated examiners (kappa statistics > 0.90) following WHO criteria and recommendations for oral health data collection [24]. Data collection extended from March to October 2011.

### Measures

The behavioral model of health services framework used in this study provides an arrange of variables that predict the utilization of medical, dental, and hospital services [25–28]. The model’s three domains; *predisposing variables* (demographic and attitude), *enabling variables* (e.g. income, region of residence, type of health insurance), and *need* have been found to be interrelated, and to predict personal health practices, use of professional health services, and satisfaction with one’s health.

In addition to age and sex, the following socio-demographic information was collected: Marital status classified into two groups: ‘Married or in a de-facto relationship’; and ‘Single, widow/widower or divorced’. Participants were classified according to their educational level using three categories: ‘No formal education to complete primary education’; ‘Incomplete secondary education’; ‘Complete secondary education or Post-secondary education’. While the questionnaire did not include a specific query on income-income was assessed though participants’ status for their health insurance. Specifically, participants were asked about their health insurance, in the categories of: ‘No health insurance or Government health insurance’; and ‘Private health insurance’. Municipalities were classified as: ‘Urban’ (i.e., head of regional province); ‘Non-metro centre’ (i.e., municipality of more than 20,000 inhabitants); and ‘Rural’ (i.e., municipality or location of 20,000 or less inhabitants).

Psychosocial variables included: Oral health attitudes, measured by seven items concerned with: the inevitability of oral disease in older adults, desirability of keeping natural teeth and the efficacy of preventive behaviors, such as self-examination and dental visits. The oral health knowledge index consisted of the sum of correct responses to 38 questions about knowledge of symptoms and causes of dental caries, periodontal disease and oral cancer.

During a dental examination, examiners recorded the number of teeth present, decayed tooth surfaces, and filled tooth surfaces.

Use of oral health care services was investigated by asking participants to indicate the time interval since their last dental visit, with response option [6]: ‘12 months or less’, ‘12 months to 2 years’, ‘2 to 5 years’, and ‘More than 5 years’. Dental attendance was dichotomized according to whether or not a participant had visited the dentist within the last 12 months. In addition, self-perceived barriers to oral health care were assessed by asking participants to report from a list of nine commonly described categories: ‘Cost’; ‘Non availability of dentist’; ‘Time waiting for appointments’; ‘Rude behavior from staff’; ‘Location of service’; ‘Fear of dentist/treatment/procedures’; ‘Access to health facilities’; ‘Physical disability’; and ‘General health problem’, whether any of them prevented people from accessing dental care service. Responses were coded as ‘Yes’ or ‘No’. A Barriers Index was created by adding positive responses.

Basic descriptive information on selected socio-demographic, psychosocial, dental status and use of oral health service variables were calculated. To establish whether differences existed between groups on dependent variables, ANOVAs and Chi-square tests were conducted. Logistic regression analysis was used to examine whether any combination of predisposing, enabling, need, and clinical variables, provided a multivariate explanation for oral health care services utilization. The final regression model was created using a stepwise selection method. Data manipulation and analyses were conducted using SPSS PC (Version 20.0).

## Results

Four-hundred and thirty-eight older adults aged between 65 and 74 years participated in the study. The mean age of the sample was 69.7 years (s.d. 3.4). The majority were female (77.4%) and age ranged between 70 and 74 years (52.7%). By level of education, the majority (61.0%) had complete or incomplete primary education, with 26.9% having some secondary education, including 3.2% who had tertiary education. The remaining 12.1% had no formal education. The majority was retired or indicated not working outside home (86.4%). Only 6.6% indicated that they were still working or were looking for a job (7.1%). Almost half of the sample (49.1%) was married, another 32.9% were widow/widower and 18.0% were single or divorced. The largest proportions lived in urban areas (61.6%); 20.5% lived in non-metro centres and another 17.8% lived in rural areas. The great majority (90.6%) was under public health insurance (See Table 1).

**Table 1:** Univariate associations between socio-demographic variables and use of oral health service in the previous 12 months by Chilean older adults

		Used dental service in last 12 month	
Predisposing, enabling, and need factors	n	(%) Yes	(%) No
Mean Age (years)		69.9 (3.4)^a^	69.7 (3.5)
Gender			
Male	99	35.4	64.6
Female	339	30.4	69.4
Level of education			
No formal/primary education complete	53	22.6	77.4
Some secondary education	267	33.0	67.0
Secondary education complete/some tertiary education	118	32.2	67.8
Place of residence		**	
Urban	270	29.6	70.4
Semi-urban	90	25.6	74.4
Rural	73	44.9	55.1
Health insurance			
Public	397	31.2	68.8
Other	41	34.1	65.7
Oral health knowledge		11.2 (7.1)^a^	10.6 (6.8)
Attitudes to health care*		2.8 (1.3)^a^	3.1 (1.4)
Barriers to treatment		1.4 (1.2)^a^	1.6 (1.3)
Edentulous		**	
No	333	34.8	65.2
Yes	105	21.0	79.0
Number of teeth**		12.3 (9.3)^a^	9.7 (9.9)
Missing teeth***		19.7 (9.3)^a^	22.3 (9.9)

^a^Mean and standard deviation; *p < 0.05; **p < 0.01; ***p < 0.001

Figures may not add due to missing values

Overall, 31.5% of participants reported having a dental visit in the previous 12 months. Seventy-five participants (17.1%) reported that their last visit had been more than 1 year, but less than 2 years ago. An additional 21.0% had not been to the dentist within the last 2 years, but not more than 5 years; and 30.4% had not been for more than 5 years ago. Amongst those who visited the dentist in the previous 12 months, the most common reasons for the visit were dental prostheses (35.5%), followed by tooth extraction (29.0%), or dental crown/filling (21.7%). A dental check-up was nominated by 5.0%. No differences were present by socio-demographic variables or reason for the last visit. However, those living in rural localities tended to be more likely (*p* = 0.063) to have visited for an extraction. Among those who had a dental visit in the previous 12 months, almost half nominated private oral health services (47.8%) a similar proportion nominated public services (46.4%). Another 5.7% indicated having attended a dental technician.

The three most frequently nominated barriers to care were cost of services (60.3%); difficulties obtaining dental appointments (32.2%); and fear of dentists (21.7%). Other commonly mentioned barriers were availability of oral health care services (18.0%), and access to oral health services (11.6%). No other category was reported by more than 5% of the sample. One-hundred and eighty-nine participants (43.2%) reported one barrier to oral health services; 21.5% and 12.1% reported two and three barriers, respectively. The remainder (7.3%), reported between four and eight barriers. Seventy participants (16.0%) reported no barriers.

Univariate associations between socio-demographic, predisposing, enabling and clinical variables and use of oral health care services are shown in Table 1. The analysis indicated that those who visited the dentist were more likely to have lower scores for oral health attitudes than those who did not (3.1 vs. 2.8, respectively; *p <* 0.05). By place of residence, those living in rural areas were more likely to have visited the dentist (44.9%) compared to those living in urban or in non-metro centres (29.5% and 25.6%, respectively; *p <* 0.05).

Concerning clinical conditions, the mean number of natural teeth was 10.5 (s.d. 9.8). On the other hand, the mean number of missing teeth was 21.5 (s.d. 9.8). One-hundred and five (24.0%) participants were fully edentulous. Edentulous participants were less likely to have been to the dentist in the previous 12 months than those with natural teeth (34.8% vs. 21.0%; *p <* 0.01; *OR =* 0.82; 95% CI: 0.73–0.94). Also those who had visited the dentist had a significantly higher mean number of filled surfaces and missing teeth than participants who had not been to the dentist recently (6.5 vs. 3.0; and 22.3 vs. 19.7; *p <* 0.001, respectively).

The probability of using oral health care services in the last 12 months, was explored with logistic regression analysis utilizing as predictors five predisposing variables (age, sex, education, marital status, and attitude to oral health), three enabling and needs variables (place of residence, oral health knowledge and type of health insurance) and three clinical variables (filled surfaces, decayed surfaces and edentulousness). In the final model four variables remained significantly associated with use of oral health care services [*χ* ^2^(4) = 40.82; *p <* 0.0001]. Those who lived in rural areas were more likely to have visited the dentist (*OR =* 2.15; 95% CI: 1.27–2.63). Those who reported fear of dentists were less likely to have visited the dentist compared to those who did not report fear (*OR =* 0.43; 95% CI: 0.24–0.76). Participants with, at least, secondary education were more likely to have visited the dentist than those with lower levels of education (*OR =* 1.66; 95% CI: 1.03–2.64). Additionally, filled surfaces increased the likelihood of having had a dental visit (*OR =* 1.08; 95% CI: 1.05–1.11) Table 2 presents the results of the logistic regression. The variance in oral health care use explained by the final model was 12.5% (Nagelkerke *r* ^2^ = 0.125).

**Table 2:** Regression coefficient, odds ratios and 95% confidence interval for odds ratios for the factors predicting use of oral health care services among Chilean older adults

	β coefficient	Odds ratio	95% Confidence interval
Place of residence			
Rural	0.77	2.15	1.27–3.63
Urban/Semi-urban			1.00
Fear to dentist (Yes =1)	−0.85	0.43	0.24–0.76
Level of education			
Secondary complete and higher levels	0.50	1.65	1.03–2.64
Less than secondary complete			1.00
Filled tooth surfaces	0.73	1.08	1.06–1.11
Constant	−1.41		

The variance in dental visits accounted for using the full model was 12.5% (η^2^ = 0.125)

## Discussion

Present results indicate that 31.5% of older Chilean adults participating in this study had a dental visit within the 12 months prior to the survey. This represents a somewhat lower proportion to that found in the Chilean data (33.6%) for people 65 years and older in the Valparaiso Region in 2007 and national data in 2010 (40.4%) [16, 17]. More importantly, although this was a largely dentate sample, about a third of this group (30.4%) reported not having a dental visit for 5 years or longer. This is even more significant considering that the majority of the sample had access to emergency and general oral health care through state own health services and, for those 60 years-old, access to comprehensive oral health care through the GES [3, 29]. Thus, access to public oral health care for older adults is based on whether or not they meet restrictive age criteria, or compete for access with other age groups on a “first-come first-served” basis. In Chile, approximately 23% of the 18,115 dentists work in the public system to cover 85% of the population. This represent a 40% deficit in the public versus the private system, which leaves this age group with not many choices apart from private care [30].

The results from logistic regression analysis showed that there were significant disparities in oral health services utilization by level of education, place of residence, and clinical outcomes, such as filled surfaces. These results are partly consistent with previous findings with respect to socio-demographic status (e.g., level of education). On the other hand, place of residence effects were not in the expected direction. In the present study, those living in rural areas were more likely to use oral health care services than those who lived in urban or non-metro centres. Previous studies commonly report lower use of services by rural residents [5, 31, 32]. An explanation could be that GES, may have changed oral health behaviors in this population, which may reflect public awareness campaigns. Thus, reconfirming the conclusion that the GES has improved services utilization among those who traditionally are found to have greater difficulties accessing oral health services (e.g., rural).

However, this increase in frequency does not translate into better oral health status, as demonstrated in participants’ periodontal and oral health status, and use of prosthetic appliances [2]. Furthermore, there was a trend for those living in rural areas to have less conservative interventions, which suggests that interventions aimed at closing the gap in oral health services utilisation need more attention.

As demonstrated in the present study, use of oral health care services among older adults is affected by many socio-demographic, and psychosocial factors, as well as other predisposing and enabling factors. Participants identified several commonly reported barriers to accessing oral health care services (e.g., cost, availability of oral health care services, length of waiting lists, and fear of dentist) [1, 11, 21, 33], as impacting on their use of oral health services. Nonetheless, fear was the only barrier that remained significant in the multivariate model. Participants who reported fear of dentist or treatments, were less likely to have attended the dentist.

These results have important program and policy implications, as in order to increase use of oral health care services in this population, additional efforts, beyond the reduction of costs or financial barriers, would be required. The results support arguments for increasing the awareness of the need to periodically visit a dentist, and fear reduction and desensitization, at any age. This should be viewed as an appropriate and socially just intervention. Still, further is required to properly establish the relationship between rurality and oral health care services utilization. For example, no attempt was made to collect information on the participants’ oral health visit experience, perspectives and influences using qualitative or mixed methods approaches. These data would have provided a better and richer appreciation of older adults’ perception and ideas. In making these observations, the limitations of the study design have to be considered within. This was a sample of volunteers recruited from older persons clubs in the Maule Region who were healthy, reasonably well and autonomous, to be able to independently manage their activities and to participate in social clubs. Additionally, data were based on self-reports, and the potential for recall and social desirability biases on the frequency of visits, might also be present. Third, the data was from a regional representative sample. As such, it provides information at a regional level. The findings are not intended to be generalised to all older Chileans. Fourth, there was no differentiation between dental visits. A basic variation would be between emergency and general dental care visits. Another limitation was the socio-economic variables employed in the study may be too limited, other important predictors such as actual income, social support, or oral health literacy were not investigated.

Furthermore, although the final multivariate model provided a significant description of oral health service use, the final model accounted for 12.5% of the variance, only. Thus, predictors not considered in this study might have added further explanatory power to the model. This might include self-assessed oral health needs and status, self-efficacy and other dental and personal considerations. Alternatively, commonly described predictors might not directly apply to older Chilean populations. Also, it may be that those predictors need to be questioned since older adults keep more natural teeth and therefore, have different oral health needs and demands for services [1, 21]. Thus, this study does not claim that a definitive model has been identified; rather the study raises some potentially important predictors to be explored in future endeavors.

Nonetheless, while the present study may have limitations, due to the dearth of information regarding the oral health of older adults in Chile, it was considered important to conduct this analysis. The purpose of the study was to gain an initial understanding of, and identify factors associated with, the use of oral health services among older Chileans, and to provide relevant information for the design of future studies aimed at determining predictors of their use oral health service. Moreover, the study also has several strengths. As the proportion of edentulous people decreases, there is an increased need for oral health care services. This study contributes to a better understating of the oral health care visits of community-dwelling older adults living in the Maule Region of Chile. We explored the effect of place of residency on oral health services utilization, which may have important implications for other researchers. It is noteworthy that the direction of the rurality effect on the use of oral health care services, after adjusting for the other commonly used SES indicators, suggests the need for further understanding of the pathways that lead to the disparity in oral health. Rural communities have an impressive number of strengths and assets [33]. An improved understanding of factors related to the use of oral health care services by older adults living in urban, rural and non-metropolitan centers will also need to be met.

Future research should build on these preliminary insights and better explore the source of these discrepancies regarding predictors of the use of oral health care services described in the literature, better assess the capacity of older adults to manage their health problems, and make a distinction between types of visits. As the urban population is increasing in Chile, it is important to explore these effects with respect to other health outcomes. An equitable use of health care is an important goal within a health care system [29].

## Conclusion

The study highlighted the need for additional exploration of the oral health care visit behaviors of older adults. Learning how to reach these groups and overcoming barriers to care are matters of increasing interest, given the growing size of this segment of the population and the relevance of oral health within the broad concept of oral health and wellbeing. Using information provide in this study, health planners could better develop programs and policies to address disparities in access to both preventive and restorative oral health services for all Chileans.

## Additional files

Additional file 1: Oral Health Interview. English copy of the questionnaire/survey guide used in this study. (DOCX 126 kb)

## Abbreviations

ANOVA: Analysis of variance
CI: Confidence interval
GES: Explicit health guarantee
OR: Odds ratio
SES: Socio-economic status
WHO: World Health Organization
