# Supplementary material for: Patterns of use of oral health care services and barriers to dental care among ambulatory older Chilean
Source: BMC Oral Health. 2017 Jan 9;17:38. doi: 10.1186/s12903-016-0329-2 (PMC5223417; doi:10.1186/s12903-016-0329-2)
Supplement: Additional file 1: — Oral Health Interview. English copy of the questionnaire/survey guide used in this study. (DOCX 126 kb) [file 12903_2016_329_MOESM1_ESM.pdf]

ID No.: ____________

Oral Health Interview

1 Interviewer ID ______________________________________

2 Age (years): ____________

3 Sex Female ①
Male☐ ②

4 Municipality ______________________________________

TELL ME HOW YOU TAKE CARE OF YOUR MOUTH

Which of the following oral health practices did you perform yesterday?

(Check **all** that apply; *mark one circle on each line)*

		yes	no
**5**	Brushing	①	②
**6**	Flossing	①	②
**7**	Use of wood stick	①	②
**8**	Use of toothpicks	①	②
**9**	Mouthwashes	①	②
**10**	Others. Please, specify ___________________________	①	②

11 **How often do you brush your teeth?** *(Mark one circle only)*

① Once a day
② 2 or more times a day
③ 3 times or less a week
④ At least once a week
⑤ Less than once a week
⑥ Never

12 **How often do you use dental floss?** *(Mark one circle only)*

① 2 or more times a day
② Once a day
③ 3 times or less a week
④ At least once a week
⑤ Less than once a week
⑥ Never

13 How much oral health information do you receive? *(Mark one circle only)*

① Too much
② Right amount
③ Not enough

Please indicate where have you seen or heard information about oral health

	(Check **all** that apply; *Mark one circle on each line)*	Yes	No
14	TV	①	②
15	Radio	①	②
16	Newspaper, magazines	①	②
17	Health brochures	①	②
18	Friends, relatives, neighbors	①	②
19	Health Clinics, medical centers,	①	②
20	Street advertisement, in trains, buses, etc.	①	②
21	Other, specify	①	②

During the LAST 6 MONTHS, how often have you experienced the following:

		Never	Seldom	Sometimes	Often	Very often	Always
22	How often did you limit the kinds or amounts of food you eat because of problems with your teeth or dentures?	⓪	①	②	③	④	⑤
23	How often did you have trouble biting or chewing any kinds of food, such as firm meat or apples?	⓪	①	②	③	④	⑤
24	How often were you able to swallow comfortably?	⓪	①	②	③	④	⑤
25	How often have your teeth or dentures prevented you from speaking the way you wanted?	⓪	①	②	③	④	⑤
26	How often were you able to eat anything without feeling discomfort?	⓪	①	②	③	④	⑤
27	How often did you limit contacts with people because of the condition of your teeth or dentures?	⓪	①	②	③	④	⑤
28	How often were you pleased or happy with the looks of your teeth and gums, or dentures?	⓪	①	②	③	④	⑤
29	How often did you use medication to relieve pain or discomfort from around your mouth?	⓪	①	②	③	④	⑤
30	How often were you worried or concerned about the problems with your teeth, gums, or dentures?	⓪	①	②	③	④	⑤
31	How often did you feel nervous or self-conscious because of problems with your teeth, gums, or dentures?	⓪	①	②	③	④	⑤
32	How often did you feel uncomfortable eating in front of people because of problems with your teeth or dentures?	⓪	①	②	③	④	⑤
33	How often were your teeth or gums sensitive to hot, cold, or sweets?	⓪	①	②	③	④	⑤

THINKING ABOUT ORAL HEALTH

In your opinion, what are some of the warning signs of tooth decay (dental caries)?

(Mark one circle on each line)

		Yes	No	Do not know
34	Pain in the tooth under hot or cold (e.g. food, water	①	②	③
35	Pain in the tooth when eating sweets	①	②	③
36	Continuous pain	①	②	③
37	Change of colour of tooth to darker shade	①	②	③
38	Hole in tooth	①	②	③
39	There are no warning signs	①	②	③

In your opinion, what are some of the warning signs of gum (or periodontal) disease?

(Mark one circle on each line)

		Yes	No	Do not know
40	Pain in the gums	①	②	③
41	Pain in the teeth	①	②	③
42	Bad breath	①	②	③
43	Bleeding gums	①	②	③
44	Swelling gums	①	②	③
45	Loose tooth	①	②	③
46	Change of colour of gums	①	②	③
47	There are no warning signs	①	②	③

In your opinion, what are some of the warning signs of oral cancer?

(Mark one circle on each line)

		Yes	No	Do not know
48	Pain	①	②	③
49	Sore or ulcer in mouth or lips	①	②	③
50	White/red patch in the mouth that does not go away	①	②	③
51	Tongue or jaw movement become difficult	①	②	③
52	There are no warning signs	①	②	③

After I read each of the following, tell me if you think it is very important, probably important, probably not important, or definitively not important in preventing dental caries (tooth decay)?

	How important is:	Very important	Probably important	Probably not important	Definitively not important	Do not know
53	Brushing teeth	①	②	③	④	⑤
54	Use dental floss	①	②	③	④	⑤
55	Drink fluoridated water	①	②	③	④	⑤
56	Use fluoridated toothpaste	①	②	③	④	⑤
57	Use dental sealant	①	②	③	④	⑤
58	Cut down frequency of eating sweets	①	②	③	④	⑤
59	Make regular dental visits	①	②	③	④	⑤

Now, I am going to ask about preventing gum disease (periodontal disease). How important or not important is each of the following statements in preventing gum disease?

	How important is:	Very important	Probably important	Probably not important	Definitively not important	Do not know
**60**	Brushing teeth	①	②	③	④	⑤
**61**	Use dental floss	①	②	③	④	⑤
**62**	Drink fluoridated water	①	②	③	④	⑤
**63**	Use fluoridated toothpaste	①	②	③	④	⑤
**64**	Use dental sealant	①	②	③	④	⑤
**65**	Cut down frequency of eating sweets	①	②	③	④	⑤
**66**	Make regular dental visits	①	②	③	④	⑤

Now, I am going to ask about mouth or lip cancer.How important or not important is each of the following statements in causing mouth or lip cancer?

	How important is:	Very important	Probably important	Probably not important	Definitively not important	Do not know
**67**	Heredity	①	②	③	④	⑤
**68**	Spending time under the sun	①	②	③	④	⑤
**69**	Bad nutrition	①	②	③	④	⑤
**70**	Ageing	①	②	③	④	⑤
**71**	Smoking	①	②	③	④	⑤
**72**	Poorly fitted full or partial denture	①	②	③	④	⑤
**73**	Excessive drinking of alcohol	①	②	③	④	⑤
**74**	Regular use of both alcohol and tobacco	①	②	③	④	⑤

More About ORAL HEALTH ...

For the following items, please tell me how much do you agree or disagree with each of the following statements.

Remember there is no right or wrong answer, we are interested in your opinion

		**Strongly agree**	**Agree**	**Disagree**	**Strongly disagree**
**75**	There is a lot an older person can do to prevent oral disease	①	②	③	④
**76**	Older adults have to learn to live with the condition of their teeth and gums	①	②	③	④
**77**	As people become older, they can expect to lose their teeth	①	②	③	④
**78**	Getting dentures is better than keeping your own teeth	①	②	③	④
**79**	Only dental professionals can examine my mouth for any sign of cancer of the mouth.	①	②	③	④
**80**	Having dental problems is a normal part of aging	①	②	③	④
**81**	Only people with their own teeth need to see the dentist	①	②	③	④

TELL ME ABOUT YOUR USE OF ORAL HEALTH CARE SERVICES

82 In the last 12 months did you have and oral health problems for which you would have wanted to see a dentist?

① No
② Yes
③ Yes and visited

83 When did you last visit a dentist? *(Mark one circle only)*

① 12 months or less
② More than 12 months, but less than 2 years
③ 2 years, but less than 5 years
④ 5 years, but less than 10 years
⑤ 10 years or more
⑥ Never been to a dentist in Australia
⑦ Never been to a dentist in my life
⑧ Do not know

84 The main reason for your last visit to the dentist was?

① Tooth extraction
② Tooth cleaning
③ Relief of pain
④ Fluoride application
⑤ Dental crown or Filling(s)
⑥ Denture (Construction, fitted, repaired, relined, adjusted)
⑦ Soft tissues consultation (Sore or ulcer in lips, tongue, etc)
⑧ Gum treatment
⑨ Check up only
⑩ X Rays
Other treatment . Please, specify ________________________________

85 Where did you go for your last visit? *(Mark one circle only)*

① Community Health Centre
② Private practice
③ Dental technician
④ Other. Please, specify ________________________

Have any of the following stopped you seeking oral health services?

(Check __all__ that apply; mark one circle on each line)

		Yes	No
86	Cost	①	②
87	Availability of dentists	①	②
88	Time waiting for appointments (waiting lists)	①	②
89	Rude behaviour from dentists	①	②
90	Rude behaviour from non-professionals (receptionist, dental nurses)	①	②
91	Location of services (transportation)	①	②
92	Waiting time in surgery	①	②
93	Fear of dentist/treatments/procedures	①	②
94	Physical disability (access)	①	②
95	General health problem	①	②
96	Communication/language problems	①	②

97 Marital status?

① Single
② Married
③ De-facto relationship
④ Divorced/separated
⑤ Widow/widower

98 Are you covered by any Health Insurance?

① National Health Fund
② Private Health Fund (ISAPRE)
③ Force Armed
④ Other

99 What is the highest level of education you reached? *(Mark one circle only)*

① No formal education
② Primary incomplete (year 1-6)
③ Primary complete
④ Secondary incomplete
⑤ Secondary complete
⑥ Tertiary education (complete or incomplete)

100 What is your current occupational status?

① Dependent worker (employee)
② Independent worker (self-employed, entrepreneur)
③ Unemployed
④ Retired
⑤ Not working outside home
⑥ Not actively seeking for work. Specify, ______________________________

Have we missed anything?

If there ANY OTHER ISSUES that you would like to comment on, please tell me and I will write it down?

____
____
____
____

Thank you very much for your time and effort in completing this questionnaire.

Your efforts are greatly appreciated.
Please remember that we guarantee confidentiality
and we will not read your name to anyone.
